# Airflow ejection-wrapped clamping type seedling picking method and parameter optimization

**DOI:** 10.3389/fpls.2022.1084563

**Published:** 2023-01-12

**Authors:** Guoxin Ma, Xi Chen, Yang Liu, Luhua Han, Hanping Mao, Jianping Hu

**Affiliations:** ^1^ School of Agricultural Engineering, Jiangsu University, Zhenjiang, China; ^2^ Xinjiang Academy of State Farms, Institute of Mechanical Equipment, Shihezi, China

**Keywords:** seedling picking, airflow ejection, wrapped clamping, end-effector, coupling simulation, test

## Abstract

Since the current clamp-type and push-out-type seedling picking method brought damage to seedlings, this study aimed to proposed an airflow ejection-wrapped clamping type seedling picking method, which used airflow to eject out seedling and the seedlings were wrapped clamped to reduce the damage of seedlings during seedling picking process. The parameter model was established through theoretical design, then the parameters were optimized through coupling simulation analysis, and the validity of these parameters was verified through experiments. We found that the diameter of the airflow nozzle was selected as 3.5 mm to match with the drainage outlet of the plug tray, and the airflow pressure which could eject out seedlings was calculated as 0.146 Mpa~0.315 Mpa on the basis of gas jet dynamic. The fluid-solid coupling simulation of airflow ejection in Comsol proposed that the seedlings could be ejected out under the airflow pressure was equal to or greater than 0.4 Mpa, and the airflow should be maintained for about 0.3 s to ensure the posture of the seedlings ejected out for better seedling clamping. The further fluid-discrete body simulation of airflow ejection by using Fluent-Edem coupling method indicated that the seedling was damaged under airflow pressure of 0.5 MPa, so the airflow pressure should be set as 0.4 MPa during seedling ejection process. Besides, a wrapped clamping type effector which clamped the seedlings from all sides in the form of flexible package was also designed to match with the airflow ejection method, and the RecurDyn-Edem coupling simulation showed that the end-effector could tightly clamp the seedling without damage when the angle between the clamping slices and the vertical direction was 8.5°. Finally, the airflow ejection-wrapped clamping type seedling picking device was manufactured, and the verification tests verified the simulation results. This research can provide some references for the automatic seedling picking technology.

## Introduction

China has the highest vegetable production and consumption in the world now, with the vegetable planting area of 20 million hectares and an annual output of more than 750 million tons (China National Bureau of Statistics, 2021). As a key link in the development of vegetable industry, seedling cultivation and transplanting can effectively improve the yield and quality of vegetables, which will bring great comprehensive benefits to the vegetable industry (Ma et al., 2020a). With the aging of the population and the increase of labor cost, many scholars designed semi-automatic transplanters to transplant vegetables (Kumar and Raheman, 2011), but these semi-automatic transplanters still require a lot of manual work to realize seedling picking and dropping, which will lead to low work efficiency during transplanting process (Zhang et al., 2011), thus it is necessary to develop automatic transplanters with high-speed to replace the manual work during transplanting.

As an important part to realize automatic transplanting, the pick-up device should ensure the success rate of seedling picking, with all the parts operating fast and accurately (Jin et al., 2018). The automatic pick-up device is actually composed of the end-effectors and other executive parts, as the core of the automatic pick-up device, the end-effectors must ensure the seedlings can be picked from the plug tray and dropped into the transplanting cup successfully (Ye et al., 2019). Many scholars had paid a lot of effort in exploring high-efficiency automatic seedling picking methods, which could realize automatic seedling picking instead of manual work (Han et al., 2019b). Different from the previous methods used by the semi-automatic transplanters, current automatic pick-up devices mainly adopted clamp-type seedling picking method or push-out-type seedling picking method (Wen et al., 2021).

Because of the high similarity with the manual seedling picking method, the research on the clamp-type seedling picking method was earlier, which had become a more widely used method to realize automatic seedling picking. The clamp-type seedling picking method always used the specially designed needles to insert into the seedling pot, and overcame the adhesion between the seedling pot and the plug tray in order to pick the seedlings out from the plug tray successfully (Han et al., 2015). As early as 1990, Ting designed an effector which used sliding needle to insert the seedling pot obliquely for seedling picking (Ting et al., 1990). Chio proposed an end-effector which was mainly composed of air cylinders and picking fingers with an optimal success rate of 97% (Choi et al., 2002). In order to adapt to the plug seedlings cultivated in China, Yu developed a rotary-type end-effector with two pins to pick the seedlings along the predetermined track (Yu et al., 2015), Ma proposed a pneumatic end-effector with four pins to insert and clamp the seedlings (Ma et al., 2020b). However, with the inserting of the picking pins into the seedling pot, the clamp-type seedling picking method might cause certain damage to the root system and seedling pot, and this kind of damage happened from the inside, which reduced the success rate of seedling picking. Once the plug seedlings became overgrown or the root system was weak, the root system could not wrap the substrate well, thus the damage was more serious (Mao et al., 2019). Instead of inserting into the seedling pot, Tong designed a spade end-effector with four shovel-type pins which could insert and clamp the substrate around the seedling pot and reduce the loss of substrate to some extent (Tong et al., 2019). However, the damage still existed because the pins should also overcome the adhesion between seedling pot and the plug tray during seedling picking process.

Compared with the clamp-type seedling picking method, the push-out-type seedling picking method could reduce the damage of the seedling pots in a certain extent. As a company widely used this method on seedling picking, Ferrari in Italy created a FUTURA automatic transplanter by using steel needle to push out the plug seedling (Ji et al., 2020), but this transplanter must use the special hard foam trays to cultivate the seedlings. In order to find the relationship between the steel needle and the seedling pot, Gao simulated and analyzed the working process of the steel needle pushing out the plug seedling by using EDEM software (Gao et al., 2017). However, the push-out-type seedling picking method also had high requirements for mechanical properties of plug seedlings, the seedling pots could be easily penetrated by the steel needle if the substrate was loosened. Besides, the drainage outlet size of some plastic plug trays in China was not uniform, so the steel needle might destroy the plug tray during the seedling push-out process.

In general, these two automatic seedling picking methods both used pure mechanical way to overcome the adhesion between the seedling pot and plug tray, which inevitably brought disturbance to the root system and the structure of the plug seedlings, and eventually led to the damage of the seedling pot during the picking process, thus affected the success rate of seedling picking. In addition, this phenomenon became more serious with the increasing of seedling picking frequency. In order to find a way to reduce the structural disturbance and the damage of the seedling pot, this paper proposed a new method which used airflow to eject the seedlings out from the plug tray. Since the seedling pot was in flexible contact with the airflow, the damage probability of seedlings could be reduced during the seedling picking process. Besides, the air nozzle device and the end-effector matched with the airflow ejection method were also designed according to the requirement, both the airflow and the end-effector parameters were optimized through simulations and experiments.

## Materials and methods

### Feasibility analysis of airflow ejection seedling picking method

**Figure 1:**
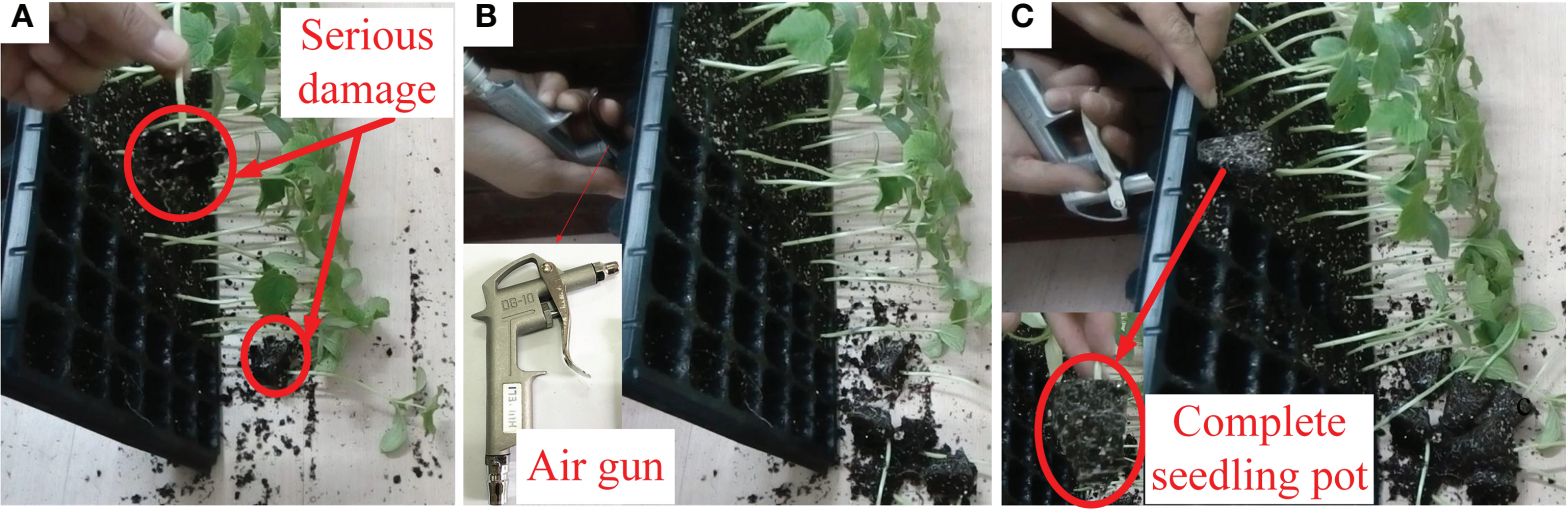
The extraction of the overgrown cucumber seedlings. **(A)** Seedlings seriously damaged after pulling out **(B)** Overgrown cucumber seedlings, these seedlings encountered continuous overcast and rainy days during cultivation, so the stem of these seedlings was long and thin, with a height of more than 200 mm. The roots of these seedlings were short and weak which could not effectively wind and wrap the substrate, so the substrate was loose and easily damaged, which led to a poor mechanical property of the seedling pots, and these seedlings could not be picked out by the conventional seedling picking methods **(C)** Seedlings remain complete after airflow ejection.

The previous research proposed that clamp-type and push-out-type seedling picking methods had high requirements for plug seedlings, the seedlings were damaged in the process of seedling picking if the strength of seedling pot was not enough, especially the overgrown plug seedlings (**Figure 1B**). As shown in **Figure 1A**, the overgrown seedling pots were seriously damaged when directly pulled out by our hand and a large amount of substrate was left in the plug tray, since the root system was poor and the substrate was loosened in overgrown plug seedling.

As shown in **Figure 1B**, a simple air gun was selected to use airflow to eject the overgrown seedlings through the drainage outlet in plug tray. As a preliminary attempt, the test result shown in **Figure 1C** was quite acceptable, the seedling was relatively complete after airflow ejection, even the overgrown seedlings with poor root systems and mechanical properties.

The internal structure of seedling pot was disturbed and the substrate was directly damaged when the clamping and push-out needles were in hard contact with seedling pot. This phenomenon was more serious if the root system of seedlings was weak, since the roots could not effectively wind and wrap the substrate to form a seedling pot with good rigidity. Besides, this type of seedling pot could not be picked out during the upward pulling process since there was a certain adhesive force between the substrate and the plug tray. Compared with the inevitable damage of needles to seedling pot, the gas, as a compressible flexible flow medium, could flexibly break through the adhesion between the seedling pot and the tray to eject the seedlings out from the tray, even the overgrown plug seedlings with poor roots and loose substrate, which reduced the disturbance to the internal structure of the seedling pot. The flow field distribution was relatively uniform when the seedlings were ejected by airflow, so the airflow pressure acted on the seedling pot was also relatively uniform, which could ensure the integrity of the seedling pot during seedling picking process. This seedling picking method had strong adaptability, which was easy to operate, and did not require high strength of the plug seedlings, so it was feasible to use airflow to eject the plug seedlings out of the plug tray.

### Establishment of airflow nozzle

The airflow nozzle device was a key component to realize airflow ejection, so the operation form of airflow and the diameter of eject hole were key factors to determine whether the seedlings could be ejected out from the plug tray.

#### Determination of the diameter of eject hole

According to the investigation of most plastic trays on the market, the drainage outlets of most plastic trays were not in the center due to the problem of injection molding process. Since the installation position and size of the airflow nozzle were determined based on the size the drainage outlet, the plug seedlings could not be ejected by the airflow nozzle when the drainage outlet was not in the standard position, thus it was necessary to select a reasonable diameter of the airflow nozzle.

**Figure 2:**
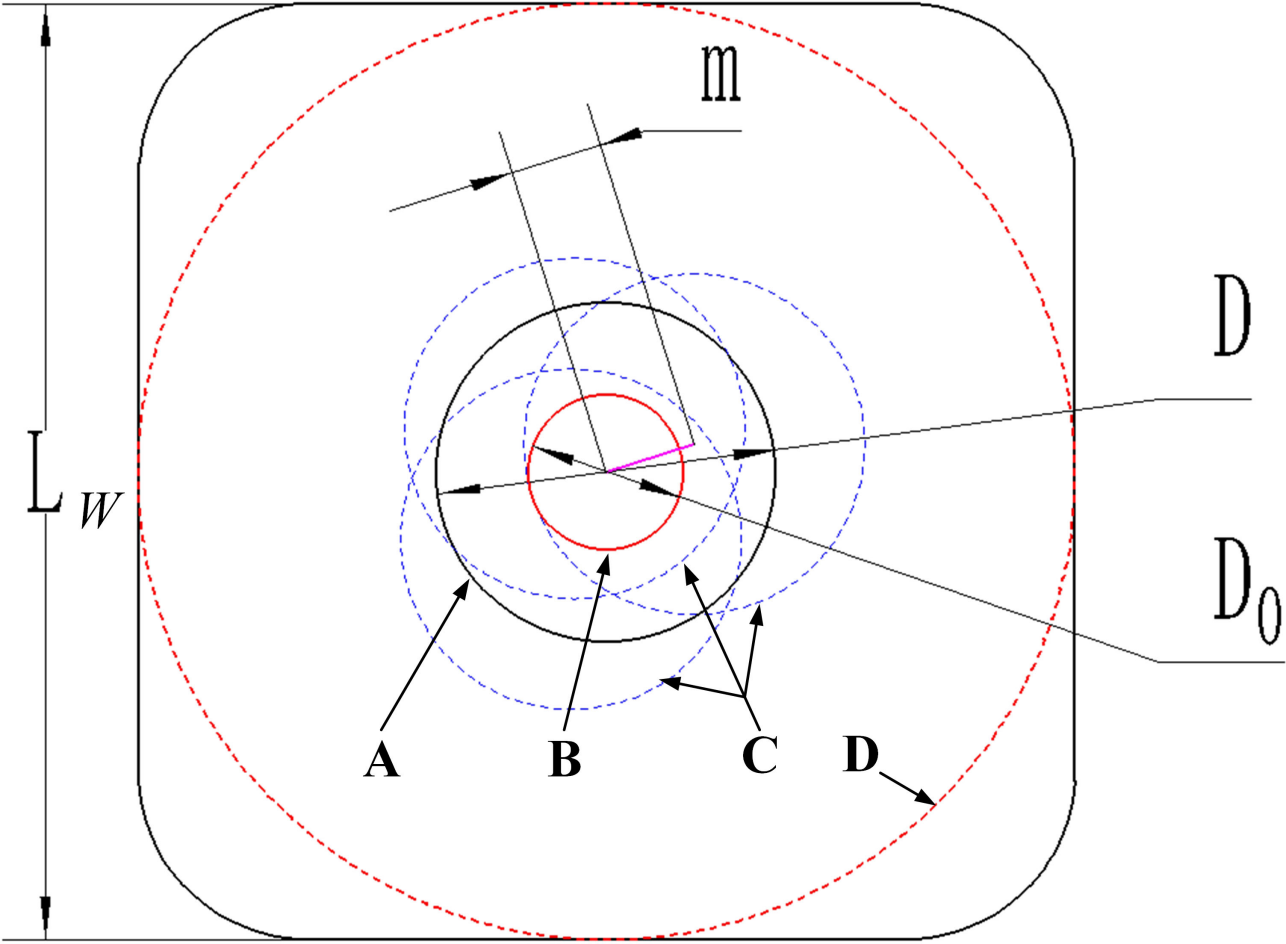
The determination of the diameter of airflow nozzle. **(A)** The standard position of the drainage outlet **(B)** The practical position of the eject hole **(C)** The possible positions of the drainage outlet **(D)** Inner diameter of the plug hole’s bottom.


The 72-hole plastic plug tray (made by Qihang Co., Jiangsu, China) widely used in China was randomly selected for measurement, with the results showed that the error of drainage outlet’s diameter was small, and the diameter was about 7 mm. Besides, the length of m marked in **Figure 2** was also measured by using simple random sampling method, and the diameter of airflow nozzle was calculated as:

(1) $$\{\begin{array}{c} {\text{D}}_{0}=2\times \left(\frac{\text{D}}{2}-\text{m}\right)\\ \text{m}=\text{max}\left({\text{m}}_{1},{\text{m}}_{2},\cdot \cdot \cdot ,{\text{m}}_{\text{i}}\right) \end{array}$$

Where *L_w_* is the width of the plug hole’s bottom, mm; *D* is the diameter of the standard drainage outlet, mm; *D*_0_ is the diameter of the eject hole, mm; *m_i_* is the distance between the center of the standard and possible drainage outlets of the *i*-th tray hole, mm; *max*() is the maximum value function.

The measurement results showed that the maximum value of *m* was 1.73 mm, so the theoretically diameter *D*_0_ of airflow nozzle should be 3.54 mm according to the calculation result of equation 1, and the diameter of airflow nozzle was finally determined as 3.5 mm according to the standard of the airflow nozzle.

#### Determination of the operation form of airflow

**Figure 3:**
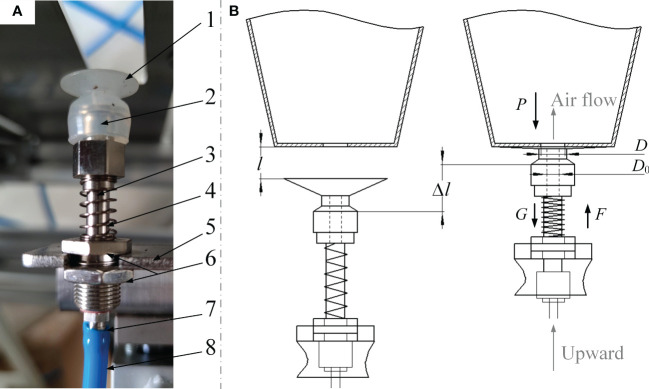
Determination of the airflow nozzle device. **(A)** The structure of airflow nozzle **(B)** Design of the pneumatic ejector device.

Since the airflow ejection method used compressed air to eject the seedlings out of the tray, the air tightness between the airflow nozzle and the bottom of the tray was particularly important. The structure of airflow nozzle was shown in **Figure 3A**, in addition to the main components, a soft sucker was installed at the front of eject hole of airflow nozzle, and a retractable ejector rod with compressible spring was also installed in order to ensure the air tightness. By the way, the outer diameter of the soft sucker should be equivalent to the inner diameter of the plug hole’s bottom (marked in **Figure 2D**).

Although the airflow nozzle needed to apply sufficient pressure to the bottom of the tray, this pressure should not exceed the ultimate pressure when the tray was about to be damaged. Therefore, we stuck the soft sucker to the bottom of the 25.4 mm cylindrical compression probe, then installed the probe on TA.XT plus texture analyzer (made by SMS Co., Godalming, Britain) to apply pressure to the bottom of the tray, so the ultimate pressure of the soft sucker to destroy the bottom of the tray was 36.15 N, so the pressure which the airflow nozzle applied to the bottom of the tray should be sufficient and should not exceed 36.15 N.

1. Soft sucker 2. Inner hole of airflow nozzle 3. Retractable ejector rod 4. Compressible spring 5. Mounting plate 6. Stop nut 7. Air inlet nozzle 8. Air tube

The pneumatic ejector device of airflow nozzle was shown in **Figure 3B**. In order to guarantee enough air tightness of the device, the cylinder should produce enough displacement to ensure that the soft sucker could attach to the bottom of plug tray, so the parameters marked in **Figure 3** should meet the following equation:


(2) $$\{\begin{array}{c} \text{l}>{\text{h}}_{1}\\ {\text{h}}_{1}<\text{y}\leq \text{l}+\Delta \text{l} \end{array}$$

Where *h*_1_ is the thickness of soft sucker after compression, mm; *l* is the distance between the upper surface of soft sucker and the bottom of plug tray, mm; *y* is the maximum stroke of ejecting cylinder, mm; *∆l* is the maximum deformation of spring after being ejected, mm.

Besides, the pre-tightening force of the spring must be greater than the reaction force of the airflow on soft sucker when seedling ejection, as:


(3) $$\{\begin{array}{c} \text{F}=\text{k}\cdot \Delta \text{l}\\ \text{F}\geq \frac{\text{π}\cdot \text{P}\cdot \left({\text{D}}^{2}-{\text{D}}_{0}^{2}\right)}{4}+{\text{G}}_{\text{d}} \end{array}$$

Where *F* is the pre-tightening force generated when the spring is compressed, N; *k* is the stiffness coefficient of spring, N/m; *P* is the downforce of airflow on soft sucker, Pa; *G_d_* is the gravity of the device, N.

Based on the pressure required by the airflow to eject the plug seedlings calculated below and the stiffness coefficient of the selected spring (18 N/m), the spring compression to ensure the air tightness of the device was at least 10.8 mm according to equation 3. Therefore, the distance between the upper surface of soft sucker and the bottom of plug tray could be appropriately reduced less than 10.8 mm or a spring with a slightly larger stiffness coefficient could be selected to obtain sufficient pre-tightening force so as to ensure the air tightness of the device.

### Feasibility analysis of airflow ejection seedling picking method

Since seedling picking was a process of overcoming the adhesion between the seedling pot and tray, the airflow pressure which acted on the bottom of seedling pot must be calculated accurately in order to ensure enough pressure to eject out seedlings.

**Figure 4:**
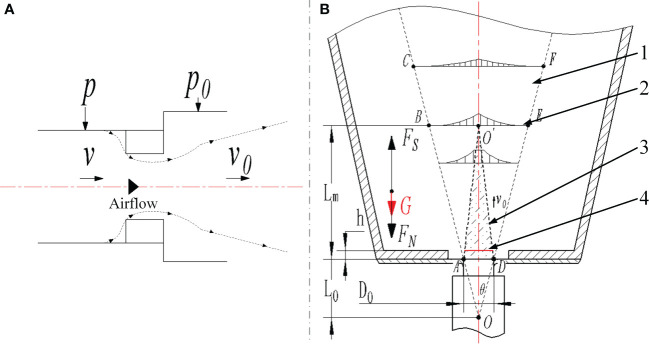
Calculation of the airflow pressure **(A)** Analysis of airflow in airflow nozzle **(B)** Analysis of airflow jet structure.


As shown in **Figure 4B**, the airflow field of the airflow acting on the plug seedling was simplified according to the gas jet dynamics (Zhao and Jiang, 1998). Since ejecting out the plug seedlings required a certain pressure, but the pressure was not the higher, the better, so the pressure should be calculated as:

(4) $$\{\begin{array}{c} F_{S}=G+F_{N}\\ F_{S}<P_{0}\cdot S<F_{B} \end{array}$$

Where *F_S_* is the force required to eject the seedling, N; *G* is the gravity of the seedling, N; *F_N_* is the adhesion between seedling pot and seedling, N; *F_B_* is the critical force for the destruction of seedling, N; *P*_0_ is the airflow pressure of airflow nozzle, MPa; *S* is the actual acting area of airflow jet, m^2^.

To ensure the stability and pressure demand of the system, 6 mm and 8 mm PVC air tubes were chose to connect the compressor and pneumatic components. Since the diameter of air tube was larger than the diameter of the airflow nozzle, it formed a shrink flow effect when the airflow passed through the nozzle (Han et al., 2004), and the airflow nozzle produced an effect as a throttle valve at this time (**Figure 4A**), so according to Bernoulli equation:


(5) $$\{\begin{array}{c} \text{C}={\left(\frac{{\text{S}}_{1}}{{\text{S}}_{0}}-1\right)}^{2}\\ {\text{v}}_{0}^{2}-v^{2}=C\cdot {\left(\frac{v_{0}+v}{2}\right)}^{2}\\ {\text{P}}_{0}=\text{P}-\frac{\text{C}\cdot \text{ρ}\cdot {\text{v}}_{0}^{2}}{2} \end{array}$$

Where *C* is the flow resistance coefficient of airflow jet; *P* and *P*_0_ are the inlet and outlet pressure of nozzle respectively, Mpa; *v* and *v*_0_ are the inlet and outlet flow velocity of nozzle respectively, m/s; *S*_0_ is the surface area of airflow nozzle, m^2^; *S*_1_ is the surface area of the drainage outlet, m^2^; *ρ* is the density of airflow, kg/m^3^.

1. Airflow jet boundary layer (DEF) 2. Airflow transition surface (BO’E) 3. Airflow jet core area (AO’D) 4. The actual area of airflow jet acting on the bottom of seedling pot *O*. The pole of airflow jet

The airflow jet core area (AO’D) marked in **Figure 4** was the main action area of airflow jet, and the airflow velocity in this area should be the same as *v*_0_ according to the gas jet dynamics, so we only needed to calculate the airflow pressure acted on the seedling. Besides, the actual area of the airflow jet acted on the bottom of seedling pot should be the red line marked 4 in **Figure 4B** since the tray had a certain thickness, so the actual acting area of airflow jet could be calculated as:


(6) $$\{\begin{array}{c} {\text{L}}_{0}=\frac{0.294{\text{D}}_{0}}{2\text{α}}\\ \text{r}=\left(1+\frac{\text{h}}{{\text{L}}_{0}}\right)\times \frac{{\text{D}}_{0}}{2}\\ \text{S}=\text{π}\cdot {\text{r}}^{2} \end{array}$$

Where *r* is the radius of actual action area of airflow jet, mm; *α* is the turbulence coefficient of airflow.

Since the selected airflow nozzle was a cylindrical copper tube, so *α* was defined as 0.076. The number of 72-hole plastic plug tray and cucumber seedlings for measurement was 20 and 72 respectively, and all of them were selected by the random sampling method, so the average thickness of the tray was measured as 0.08 mm, and the average gravity of the seedling was measured as 0.21 N. Miao proposed that the maximum detaching force of the cucumber seedling which calculated in the 72-hole plug tray was 2.011 N (Miao et al., 2013), so *F_N_* could be taken as 2.011 N. Besides, Han proposed that the critical destructive force *F_B_* of the seedling pot was 3.08 ± 0.56 N through the puncture test. By substituting the above data into equation 4, 5 and 6 for calculation and analysis, the theoretically airflow jet pressure *P*_0_ for ejecting the seedling out from the tray was between 0.146 MPa and 0.315 MPa.

The airflow ejection of the seedling belonged to an instantaneous movement, and the overall action time of the airflow was extremely short. In order to ensure the stability of the seedling ejected out from the tray for a period of time so as to ensure the smooth clamping of the seedling, it was necessary to maintain the airflow for a short period of time after seedling ejection, so the time could be calculated as:


(7) $$\{\begin{array}{c} v_{0}=\sqrt{\frac{2\cdot \left(Q-P_{1}\right)}{\rho }}\\ t=\frac{2H}{v_{0}+v} \end{array}$$

Where *Q* is the flow rate of airflow jet, m^3^/s; *P*_1_ is the difference between the inlet and outlet pressure, MPa; *H* is the height of single hole of plug tray, mm; *t* is the theoretically time of airflow jet, s.

The result showed that the total time required for airflow to eject the seedling was between 0.198 s and 0.291 s, so the maximum value was selected, and the airflow ejection time was approximately to 0.3 s in order to ensure the posture of the seedlings ejected out from the plug tray.

The airflow ejection of the seedling was analogous to the interaction between fluid and solid, while the distribution of flow field and the force acted on seedling pot were constantly changing during the process of seedling picking. Therefore, on the basis of previous calculation results, the dynamic grid module in Comsol software was selected to simulate and analyze the fluid-solid interaction of airflow ejection in order to preliminary optimize the airflow parameters. Since the damage of seedling pot was also an important factor in the process of airflow ejection, the discrete element model of seedling pot was established by Edem software, and the simulation method of Edem-Fluent fluid-discrete body interaction was established for further analysis of the effects of different airflow parameters on seedling pot particles. Finally, the prototype according to the parameters of airflow ejection seedling picking method was established, and the validation test was designed to determine the effectiveness of airflow parameters.

### Feasibility analysis of airflow ejection seedling picking method

**Figure 5:**
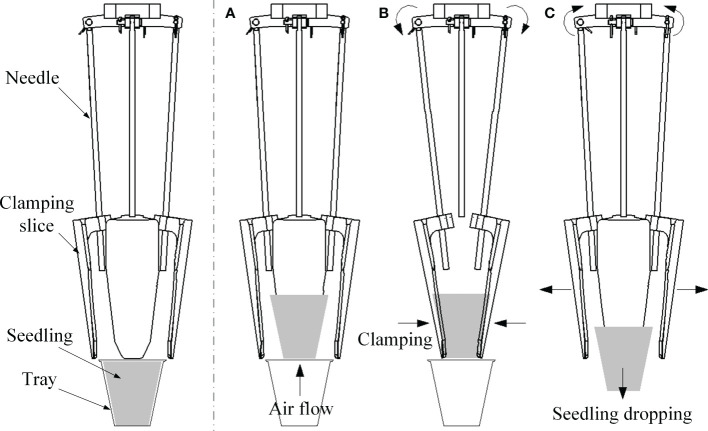
Working principle of the new end-effector **(A)** Seedling ejection by airflow **(B)** Seedling clamping **(C)** Seedling dropping.

Since the airflow ejection seedling picking method could not match with the existing end-effectors, we considered to clamp the seedling pot from all sides in the form of flexible package, which could not only ensure the smooth ejecting out of seedling, but also avoid the disturbance of root system and the damage of seedling pot during seedling clamping process. The working principle of this end-effector could be described as shown in **Figure 5**.

Before the airflow ejected the seedling out, the clamping needles rotated outward, and the clamping slices opened to a certain distance; the airflow jet ejected the seedling out from the tray to the end-effector through the drainage outlet when the airflow nozzle got the signal, and the airflow jet kept for a period of time (**Figure 5A**); the clamping needles obtained the signal and rotate inward when the seedling was ejected to end-effector, and the clamping pieces closed for a certain distance to flexibly clamp the seedling (**Figure 5B**); finally, when the end-effector was drived to the seedling dropping position, the clamping needles rotated outward again, and the clamping pieces released the seedlings to the seedling divider (**Figure 5C**).

**Figure 6:**
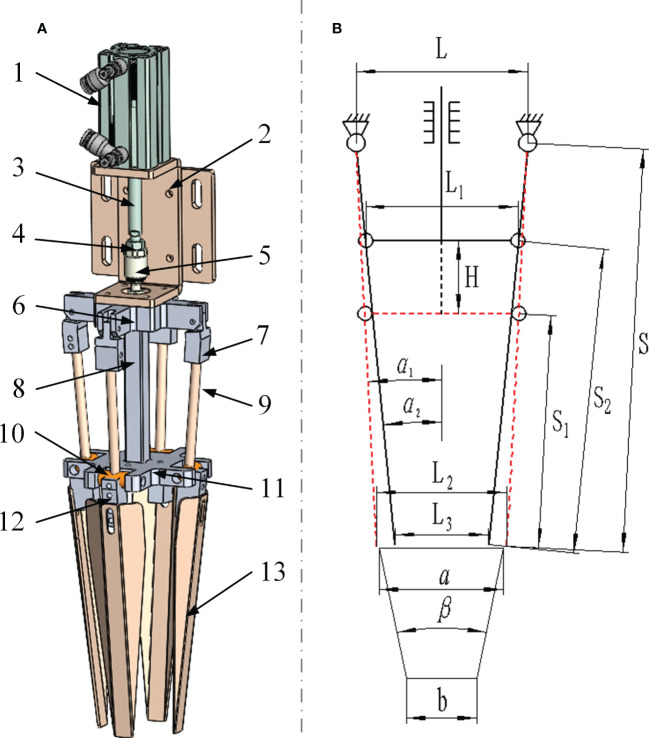
Calculation of the airflow pressure. **(A)** The new wrapped-type end-effector. **(B)** The structure diagram of end-effector.


The preliminarily designed wrapped clamping type end-effector was shown in **Figure 6A**, which was mainly composed of four rotatable clamping needles, four flexible clamping slices and a pneumatic drive device, with a simple and reliable structure. As shown in **Figure 6B**, the wrapped clamping type end-effector was simplified for calculation, the distance between the mounting points of the rotary pair at the top of the two clamping needles could be expressed as:

(8) $$\{\begin{array}{c} \text{L}={\text{L}}_{2}+2\times \text{S}\times \text{sin}{\text{α}}_{1}\\ \text{L}={\text{L}}_{3}+2\times \text{S}\times \text{sin}{\text{α}}_{2} \end{array}$$

Where *L* is the distance between the mounting points of the rotary pair at the top of the two clamping needles, mm; *L*_2_ and *L*_3_ are the tip distances when the clamping needles are opened and closed, respectively, mm; *S* is the length of single clamping needle, mm; *α*_1_ and *α*_2_ are the angle between the clamping needles and the vertical direction when the clamping needles are opened and closed, respectively, °.

1. Air cylinder 2. Mounting plate 3. Piston rod 4. Stop nut 5. Connector 6. Bracket 7. Fixture of clamping needle 8. Seedling pushing rod 9. Clamping needle 10. Dowel 11. Seedling pushing plate 12. Fixture of clamping slice 13. Clamping slice

Then the length of the seedling pushing rod could be expressed as:


(9) $$\{\begin{array}{c} L_{1}=L-2\left(S-S_{1}\right)\sin\alpha_{1}\\ L_{1}=L-2\left(S-S_{2}\right)\sin\alpha_{2}\\ H=S_{2}\cos\alpha_{2}-S_{1}\cos\alpha_{1} \end{array}$$

Where *L*_1_ is the width of seedling pushing plate, mm; *S*_1_ and *S*_2_ are the distance between the needle tip and the sliding pair of the seedling pushing plate when the clamping needles are opened and closed, respectively, mm; *H* is the movement distance of the seedling pushing plate when the clamping needles are opened and closed, mm.

Taking 72-hole plastic plug tray parameters as an example, the upper *a* and lower *b* side length of the single hole of the tray was 40 mm and 20 mm respectively, the total height of the tray was 45 mm, and the included angle between the side of the tray and the vertical direction *β* was 22.5° (Ma et al., 2020a). In addition, the range of the height of the seedling in the suitable transplanting period should be 100 to 200 mm, and the biological yield point (maximum compression amount) of the seedling pot was 3.46 mm (Han et al., 2013). Therefore, the following constraints could be obtained:


(10) $$\{\begin{array}{c} \left(b-3.46\right)mm<L_{3}<L_{2}<\left(a+3\right)mm\\ \alpha_{1}>arcsin\frac{L-\left(a+3\right)}{2S}\\ \alpha_{2}>\frac{\beta }{2}\\ S_{2}\cos\alpha_{2}>S_{1}\cos\alpha_{1}\geq 170mm \end{array}$$

Finally, combining equation 8, 9 and 10, it could be concluded that the key parameters of the wrapped clamping type end-effector were shown in **Table 1**.

**Table 1:** Key parameters of the end-effector.

Parameters	Value	Parameters	Value
*L*, mm	65.5	*S*_1_, mm	174.5
*L*_1_, mm	55	*S*_2_, mm	204
*L*_2_, mm	44.5	*H*, mm	26.4
*L*_3_, mm	20.2	*α*_1_, °	8.3
*S*, mm	225	*α*_2_, °	11.5

The optimized end-effector models were imported into the RecurDyn and Edem software respectively for RecurDyn-Edem coupling simulation in order to explore the interaction between the end-effector and the discrete element model of seedling pot in the process of seedling clamping, and finally the parameters of the end-effector were determined.

## Results and discussion

### Simulation analysis of airflow parameters

#### Fluid-solid interaction of airflow ejection

The fluid-solid interaction of airflow ejection by Comsol was shown in **Supplementary Figure 1.**

The seedling pot model was built by Solidworks, which was mainly composed of leaves, stem, seedling pot and plug tray (**Supplementary Figure 1A**), then imported into Comsol, and the parameters of seedling was defined in **Table 2**. Besides, in order to imitate the actual seedling picking state, the seedling was placed at 60° to the horizontal direction.

**Table 2:** Model parameters definition of plug seedling.

Materials	Parameters		
	Elastic modulus (MPa)	Density (kg/m^3^)	Poisson’s ratio
Seedling pot	75	243	0.5
Stem	34.92	660	0.41
Leaves	0.241	780	0.33

**Figure 7:**
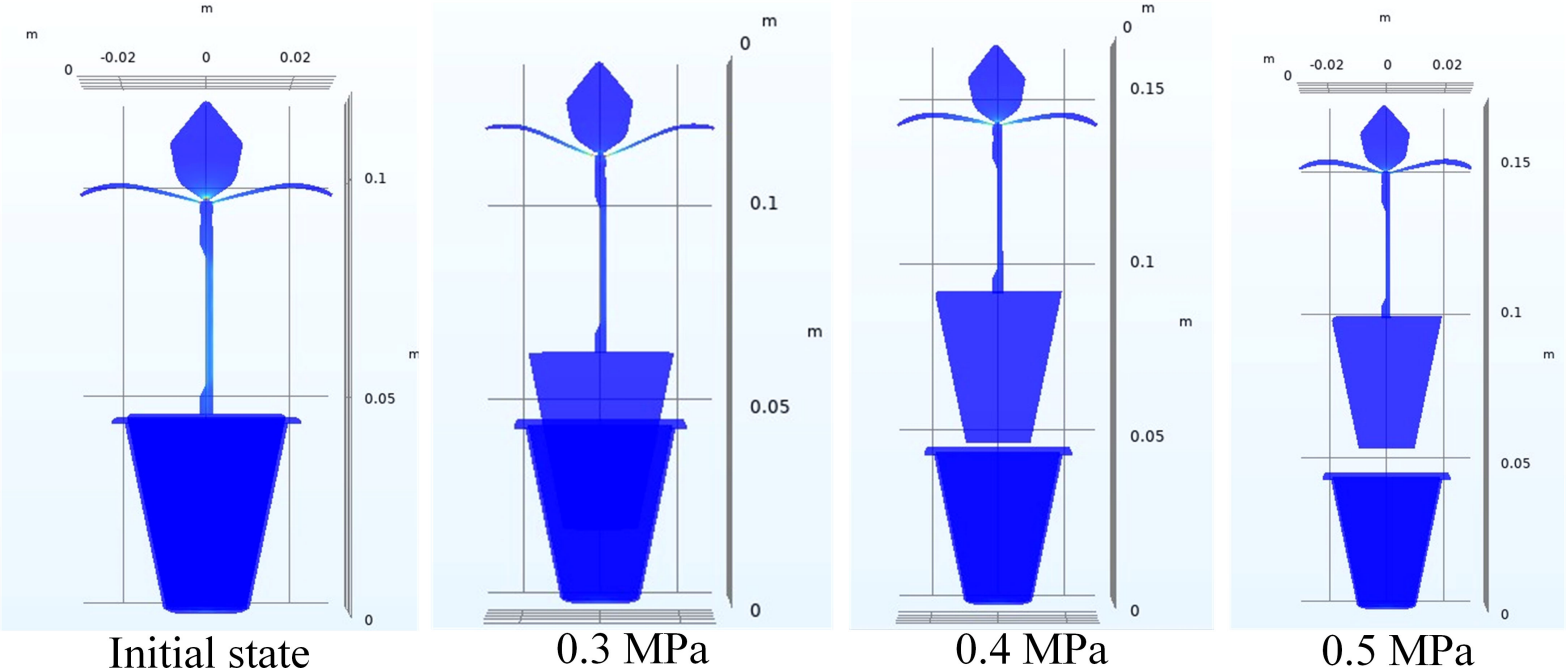
Effect of seedling ejecting under different airflow pressures.

The Physics-Controlled Mesh module was selected to grid the model, and the Moving Mesh module was used to accurately obtain the dynamic changes of seedling and airflow during the airflow ejection process. In addition, due to the transient characteristics of airflow ejection, Time Dependent method was used to divide the overall simulation process (0.3 s) into three transient calculation processes in order to improve the convergence of simulation: 0~0.1 s, because of the large calculation amount at the moment when the airflow just ejected, and the time step of this section was set as 0.001; 0.1~0.2s, the plug seedlings have been ejected out theoretically at the end of this time domain, and the time step was set as 0.01; 0.2~0.3 s, which belonged to the airflow holding phase, and the time step was also set as 0.01. After the settings were completed, the established simulation model was calculated, and the final effect of seeding ejecting under different airflow pressures was shown in **Figure 7**.

Since the maximum airflow pressure required for ejecting the seedling was about 0.315 MPa according to the calculation, considering the change and loss of airflow in the actual process, we preliminarily selected the airflow pressure of 0.3 MPa, 0.4 MPa and 0.5 MPa for the simulation test. The result showed that when the airflow pressure was 0.3 MPa, the airflow could overcome the adhesive force between the seedling pot and tray, but the seedling could not be ejected out and only moved up about 17 mm, since the airflow pressure was insufficient; however, the seedling could be ejected out when the airflow pressure increased to 0.4 MPa, since the ejection force of the airflow was large enough and the seedling could be ejected out of the tray about 2.5 mm; the seedling could also be ejected out of the tray when the airflow pressure continued to increase to 0.5 MPa, and the ejecting distance was more than 0.4 MPa, about 8 mm. The simulation showed that only when the airflow pressure was greater than or equal to 0.4 MPa, the seedling could be ejected out of the tray.

**Figure 8:**
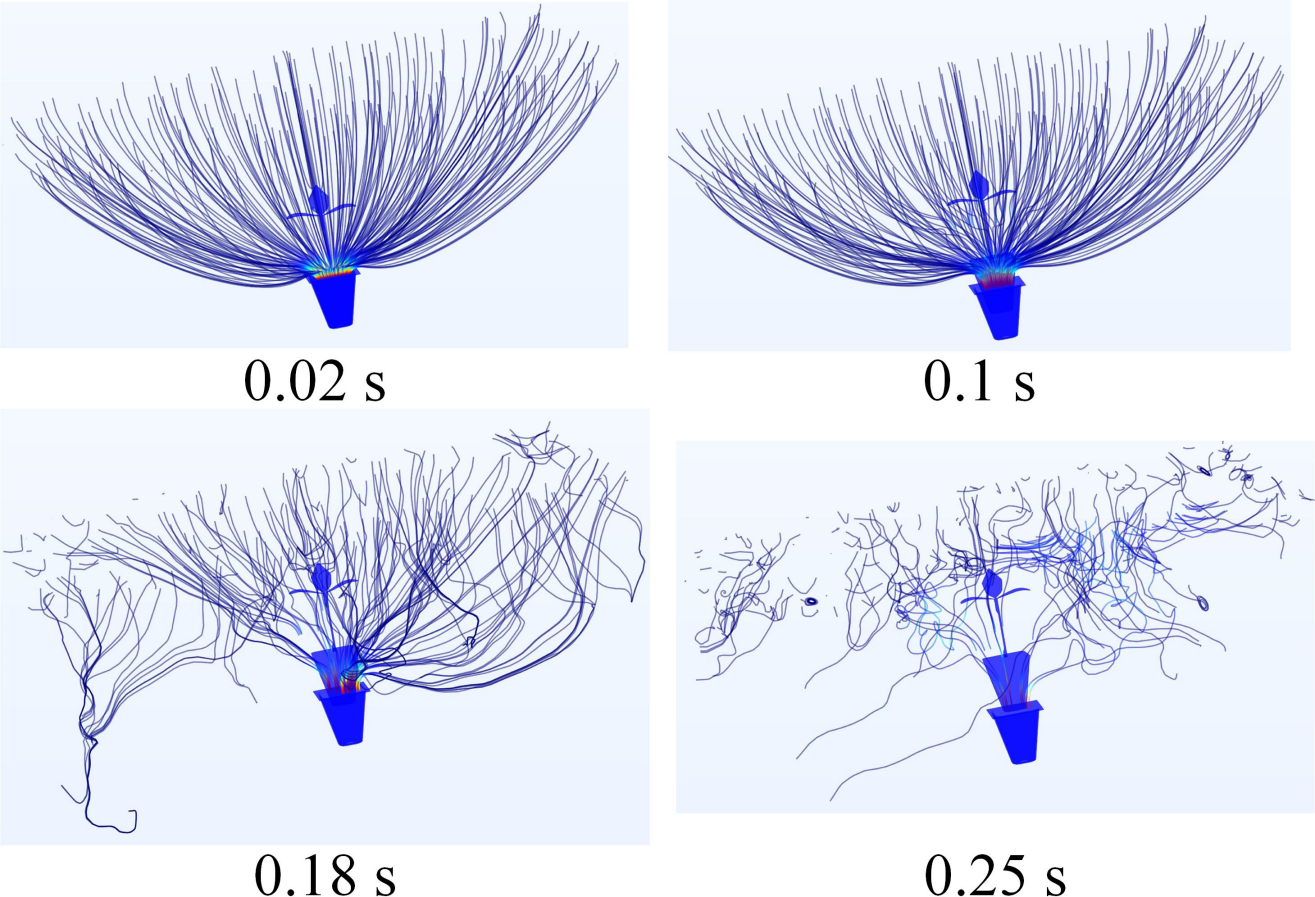
Distribution of flow field in different time periods when airflow pressure is 0.4 MPa.

The distribution of flow field in the airflow ejection process was also important. Theoretically, the seedling kept moving upward in a straight line under the action of airflow if the flow field was evenly distributed and stable. For example, the variation distribution of the flow field when the airflow pressure was 0.4 MPa was shown in **Figure 8**. The result showed that in the early stage of airflow ejection, the instantaneous force of airflow acted on the plug seedlings was large, but the flow field was very uniform and showed a divergent trend around since the moving distance of the seedling was short, so the posture of the seedling was stable. However, when the seedling was close to being ejected out of the tray, the contact volume between the airflow and the outside area was further increased, so the flow field was more disordered and irregular at this time, which might lead to changes in the posture of seedling, and this phenomenon was more serious after the seedling was totally ejected out. Therefore, it was necessary to maintain the airflow for a period of time after the seedling was ejected out in order to ensure the stability of the flow field, so as to keep the posture of the seedling being ejected out.

**Figure 9:**
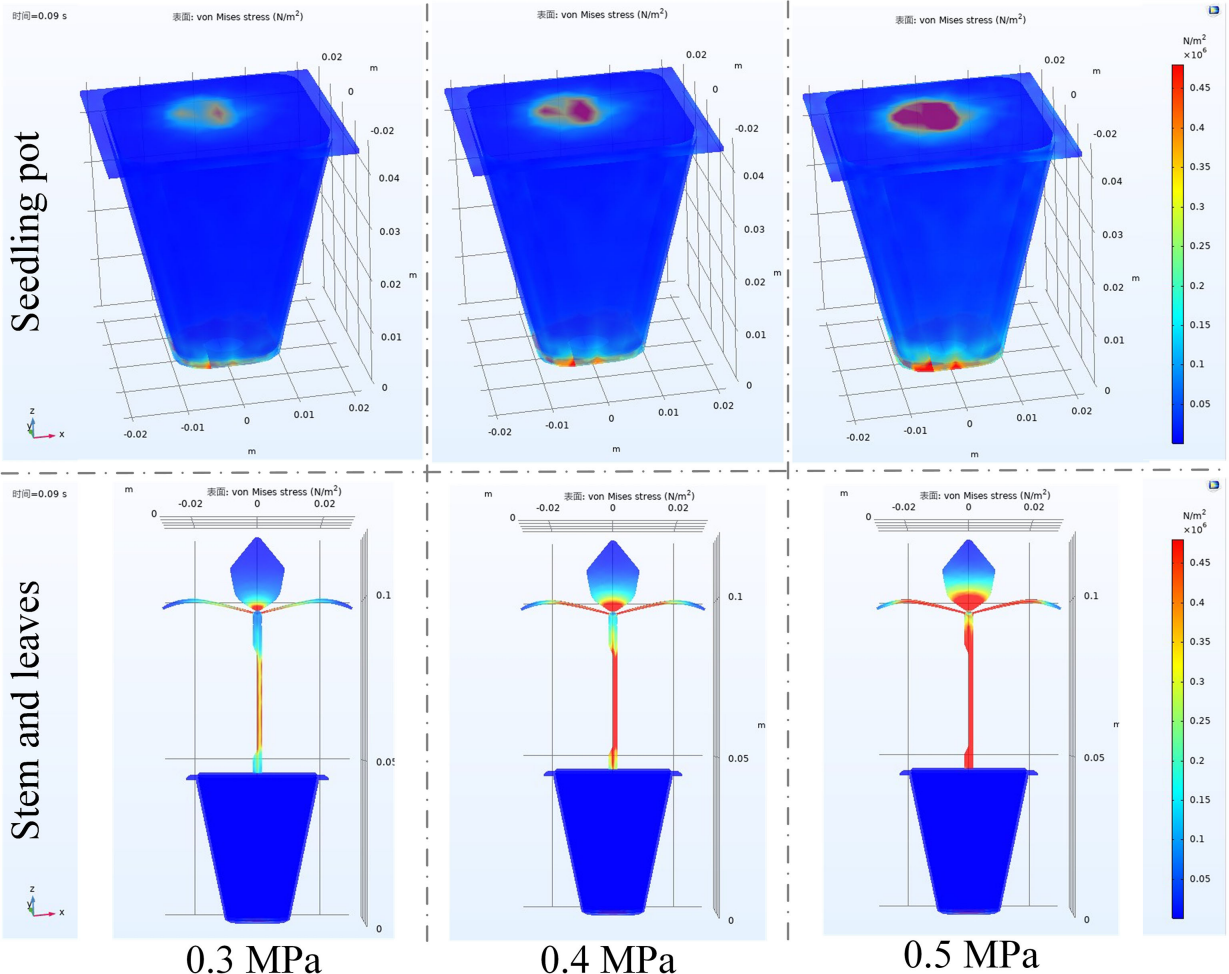
Stress analysis of seedling under different airflow pressures at 0.09s.

The stress analysis of various components in seedling under different airflow pressures was shown in **Figure 9**. The Figure showed that under different airflow pressures conditions, the main force bearing parts of the seedling were concentrated at the bottom of the seedling pot, the stem and leaves, and the area where the stem connected with seedling pot, while the stress increased with the increase of airflow pressure. Since the airflow directly contacted the bottom of seedling pot through the drainage outlet, the bottom of the seedling pot first received a direct contact force. When the seedling was ejected out, the airflow upward contacted the stem and leaves, and the flow field distribution was relatively uniform, so the force on the stem and leaf was also relatively uniform. As the airflow contacted with the stem and leaves increased, the stem and leaves were deformed to a certain extent, and the deformation also became larger with the increase of the airflow pressure. Since the width of the leaves was wide, resulting in a large surface area, the airflow produced a certain degree of pulling force to the stem, and the deformation of the stem was greater than other parts under the dual action of the airflow and the leaves. At this time, the contact force between the stem and the seedling pot was also large.

Therefore, it was important to ensure the straight growth of stem and the uniform distribution of leaves during seedling cultivation, to ensure the posture of seedling when airflow ejection.

#### Fluid-discrete body interaction of airflow ejection

On the basis of the Comsol simulation result, a discrete element model of seedling pot was established (Tong et al., 2019; Han et al, 2019a) to specifically analyze the effect of different air pressure on seedling pot particles. The Edem-Fluent fluid-discrete body interaction model was shown in **Supplementary Figure 2**, the total running time of the simulation was set as 0.3 s, and the time step was set as 0.01 s.

**Figure 10:**
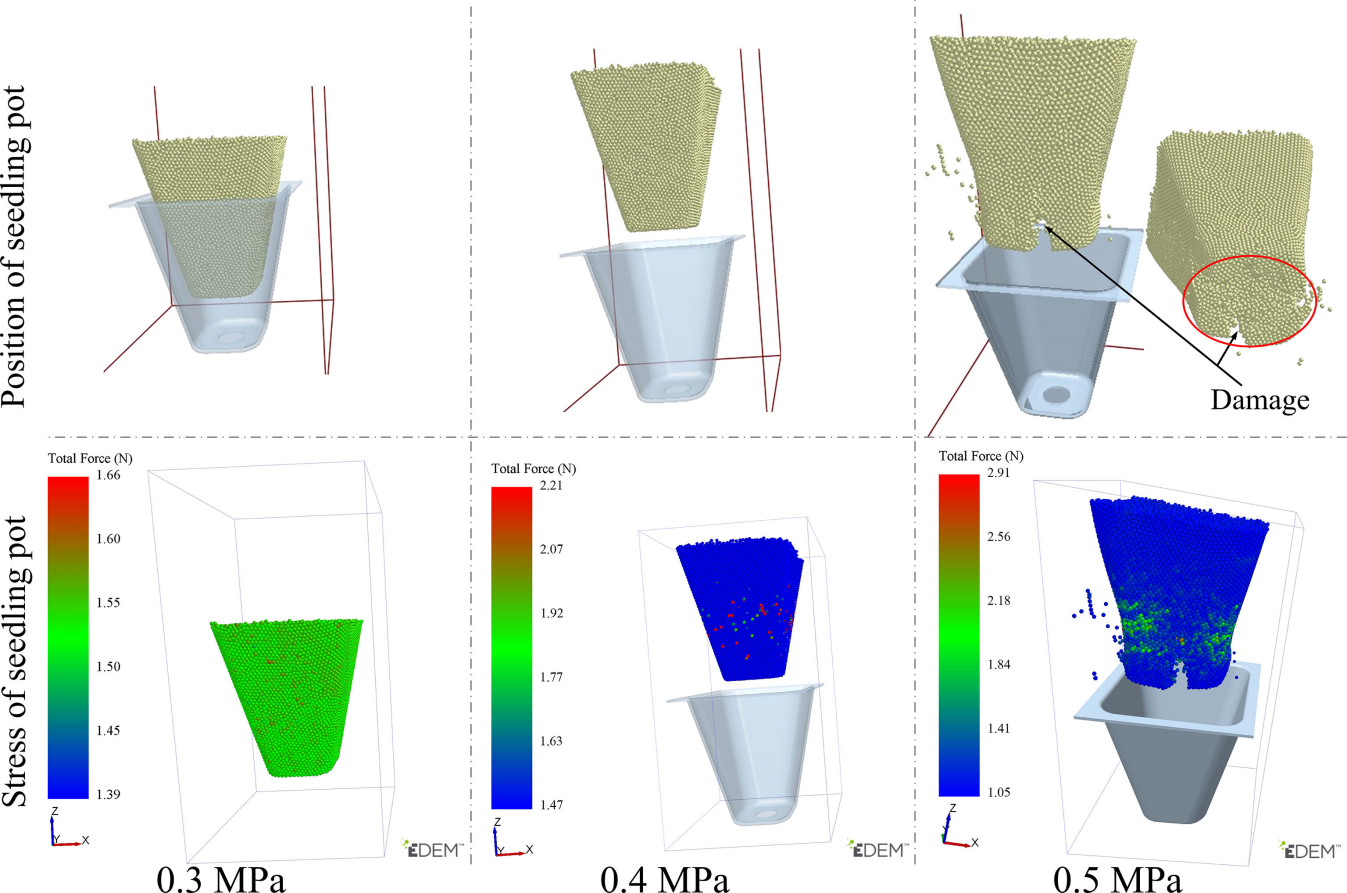
The results of fluid-discrete body interaction.

The effect of different airflow pressures on seedling pot particles was shown in **Figure 10**, consistent with the Comsol simulation results, only when the airflow pressure was 0.4 MPa or 0.5 MPa, the seedling could be ejected out of the tray. According to the statistics of the Edem post-processing module, the damage rate of bonding key in the discrete element model of seedling pot reached 43.61% and the particle loss rate reached 19.37% when the airflow pressure was 0.5 MPa.

Although the seedlings could be ejected out, the seedling pot was seriously damaged at this time. The overall damage started from the particles at the bottom which first contacted with airflow, and the damage gradually spread upward with the increase of the airflow time. When the airflow pressure was 0.4 MPa, the particles at the bottom of seedling pot also had some deformation, but this deformation was very small which only occurred when the airflow started, and the deformation disappeared when seedling pot moved up to a certain distance, so the seedling pot could not be damaged under this pressure. Therefore, it was appropriate to choose an airflow pressure of 0.4 MPa, the seedling could be ejected out of the tray about 5 mm and maintain integrity, with a vertical posture into the effective clamping range of the wrapped clamping end-effector, to achieve perfect coordination with the end-effector.

### Simulation analysis of wrapped-type end-effector

The schematic diagram of RecurDyn-Edem coupling simulation on wrapped-type end-effector was shown in **Supplementary Figure 3.**

**Figure 11:**
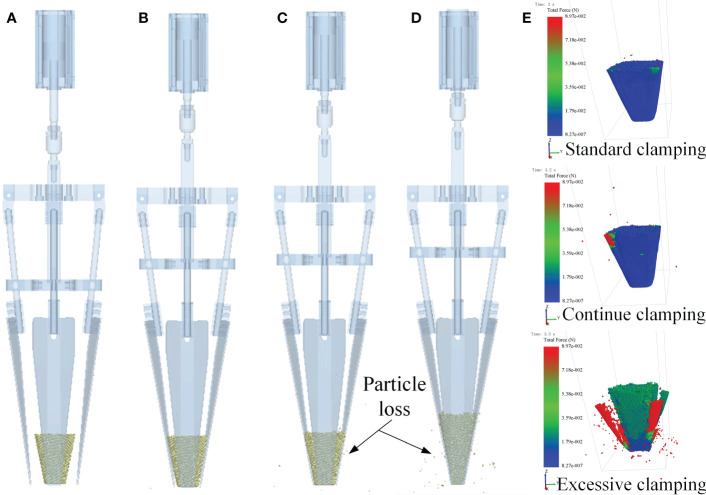
Simulation results of the wrapped-type end-effector. **(A)** Initial state **(B)** Standard clamping **(C)** Continue clamping **(D)** Excessive clamping **(E)** The process of seedling pot being clamped and destroyed.

The simulation results were shown in **Figure 11**, which proposed that the end-effector could effectively clamp the seedling pot at a certain clamping angle, with a better effect. As shown in **Figure 11B**, the clamping slice of the end-effector just contacted with the seedling pot particles, and the clamping slices only exerted a small force on seedling pot. When the clamping slices continued to close, the force of the clamping slices on seedling pot increased and the clamping effect was better. However, the force exerted by the clamping slices on seedling pot was also too large when the clamping slices closed too large, so the seedling pot was deformed, and a certain number of particles were lost (**Figure 11C**). If the clamping slices were completely closed, the seedling pot was completely damaged with the serious particle loss, and the number of bonding keys in the seedling pot model was reduced from 227969 to 104723 (**Figure 11D**). The change process of damage on the seedling pot under continuous compression was shown in **Figure 11E**. The simulation result showed that the end-effector could achieve optimal seedling clamping effect when the angle between the clamping slice and the vertical direction was 8.5°, and the clamping slice could clamp the seedling pot without deformation or damage, which was almost consistent with the previous calculation result.

### Test verification of optimized parameters

As shown in **Supplementary Figure 4**, according to the design analysis and simulation results, a simple airflow ejection seedling picking device was produced which was mainly composed of an airflow nozzle, a wrapped clamping type end-effector and an air pressure system, and the i-speed high-speed camera (made by IX Cameras Co., Essex, Britain) was selected to monitor the movement of the seedling when the seedling ejected by the airflow under different airflow pressure. The 72-hole plastic plug trays with high quality were selected for seedling cultivation, and the cucumber plug seedlings (Jinyou 1) were cultivated for 23 days under the requirements. The airflow pressure was set as 0.3 MPa, 0.4 MPa and 0.5 MPa respectively during the test process, and 20 seedlings were selected for each airflow pressure. The solenoid valve was set to be closed 0.2 s later than the original 0.3 s airflow in order to ensure the posture of the seedlings ejected out from the tray. Besides, a 0.8 MPa air compressor was selected in the test to ensure sufficient airflow supply.

**Figure 12:**
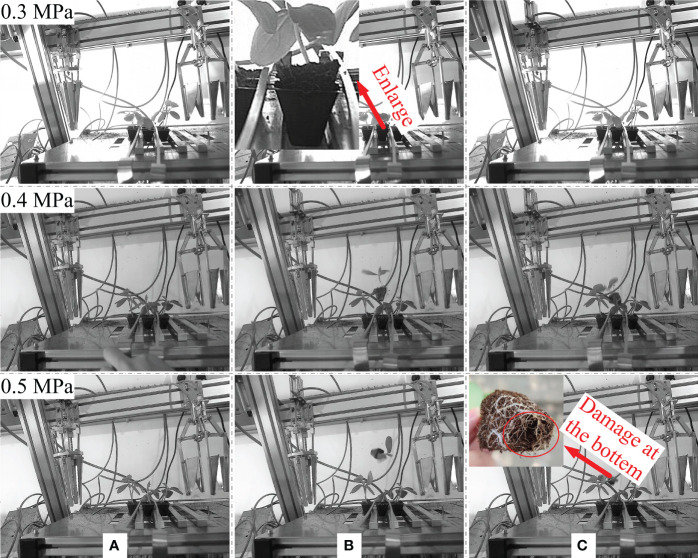
Simulation results of the wrapped-type end-effector. **(A)** Initial state **(B)** Seedling ejected out by airflow **(C)** Seedling dropping.

The airflow ejection result was shown in **Figure 12**, which showed that when the airflow pressure was 0.3 MPa, the seedling could not be ejected out of the tray, and the move up distance was only 12 mm; the seedling could be ejected out about 20 mm away from the upper surface of the tray when the airflow pressure increased to 0.4 MPa, and the posture of the seedling was also better in the process; however, when the airflow pressure continue to increase to 0.5 MPa, the seedling could also be ejected out of the plug, but the maximum distance between the seedling which was ejected out and the upper surface of the tray was more than 100 mm, with the relatively serious posture deformation of the seedling, and the seedling collided with the end-effector. Besides, the bottom of the seedling pot was damaged to a certain extent due to the excessive airflow pressure (**Figure 12C**), which was not conducive to subsequent seedling clamping. Therefore, the airflow pressure was set as 0.4 MPa, since the seedling could be completely ejected out of the tray about 20 mm away from the upper surface of the tray, without damage and with a good posture under this pressure.

**Figure 13:**
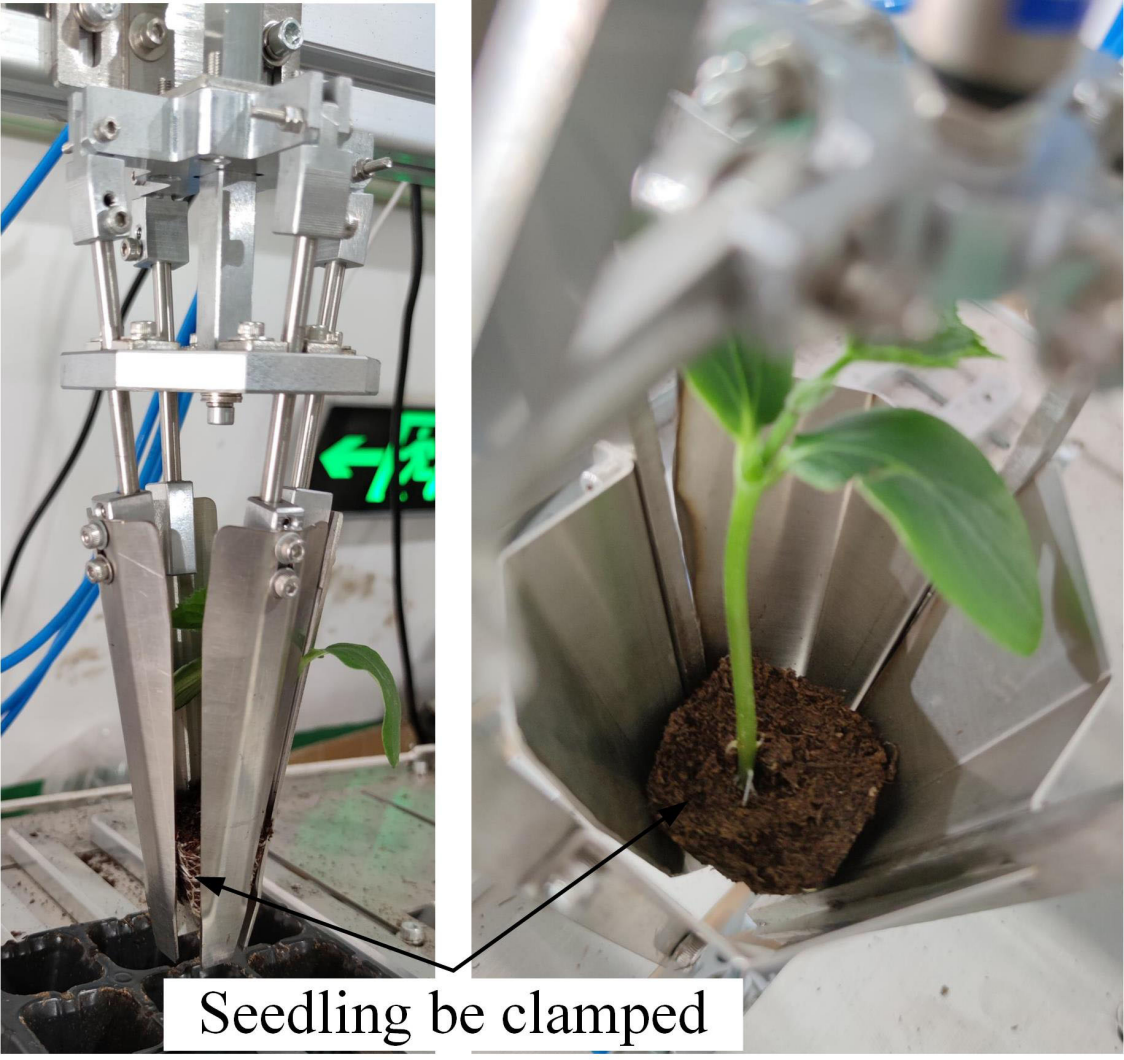
The actual seedling clamping result of new end-effector.

As shown in **Figure 13**, the test result was consistent with the simulation result. When the angle between the clamping slice and the vertical direction was 8.5° in the seedling clamping process, the seedling ejected out from the tray could be effectively clamped with a certain clamping force and without damage by the wrapped clamping end-effector. Thus, the new wrapped clamping end-effector could totally match with the airflow ejection method with a good effect of seedling clamping.

## Conclusions

Since the clamp-type seedling picking method and the push-out-type seedling picking method inevitably caused damage to the plug seedlings during the process of seedling picking, the airflow ejection-wrapped clamping type seedling picking method was proposed in order to ensure the integrity of the seedling and improve the success rate of seedling picking. The seedlings could be ejected out from the tray by the airflow and then be wrapped clamped by a new designed end-effector with low damage, which was contribute to subsequent seedling growth. Besides, both airflow ejection and end-effector parameters were optimized through simulations and experiments for a better work effect.

The operation form of airflow nozzle device was determined according to the parameters of the plug tray in order to ensure that the airflow nozzle could be tightly close to the bottom of the tray, and a soft sucker was installed in the top of airflow nozzle to guarantee the air tightness of the device. In order to eject the seedlings out from the plug tray successfully, the diameter of the airflow nozzle was calculated and finally selected as 3.5 mm, with the airflow pressure and airflow duration were calculated as 0.146 Mpa~0.315 Mpa and 0.3 s respectively according to the gas jet dynamics. Besides, the wrapped clamping type end-effector which clamped the seedlings from all sides in the form of flexible package was designed in detail according to the parameters of the plug seedlings, and the key parameters of the end-effector was optimized through mechanical design theory.

Since the airflow ejection of seedling was the interaction between the airflow and seedling, the fluid-solid coupling simulation of airflow ejection in Comsol was established to optimize the airflow parameters, and the result proposed that the seedlings could be ejected out from the tray when the airflow pressure was equal to or greater than 0.4 Mpa. Besides, if the airflow could be maintained for a period after the seedlings were ejected out from the tray, the posture of the seedlings ejected out from the tray was beneficial for seedling clamping. In order to investigate the damage of different airflow pressures on the seedling pot to further optimize the airflow parameters, a discrete element model of the seedling pot was built and the fluid-discrete body simulation of airflow ejection was established by Fluent-Edem coupling method. The results showed that the seedlings ejected out from the tray under airflow pressure of 0.4 MPa could maintain complete and the posture was good, but the seedling pot was damaged seriously when the airflow pressure was 0.5 MPa, which was not conducive to subsequent clamping. The discrete element model of the seedling pot was also used in the seedling clamping simulation by using RecurDyn-Edem coupling method, and the result showed that the seedling ejected out from the tray could be effectively clamped with a certain clamping force and without damage by the wrapped clamping end-effector when the angle between the clamping slices and the vertical direction was 8.5° in the seedling clamping process, so the new designed end-effector could match with the airflow ejection seedling picking method. The prototype of the airflow ejection-warped clamping type seedling picking device was manufactured, and the verification tests were also established, which verified the validity of theoretical calculation and simulation results. This study could provide certain guiding significance for the development of seeding picking technology.

## Data availability statement

The original contributions presented in the study are included in the article/**Supplementary Material.** Further inquiries can be directed to the corresponding author.

## Author contributions

Conceptualization, GM and HM; methodology, GM, HM, and YL; software, XC; validation, HM, JH and LH; formal analysis, GM, XC and YL; investigation, XC; resources, YL; data curation, GM; writing—original draft preparation, GM; writing—review and editing, GM, HM, JH, YL and XC; supervision, HM, JH and LH; project administration, HM, JH and LH; funding acquisition, HM. All authors contributed to the article and approved the submitted version.
