# Supplementary figures and images for: Airflow ejection-wrapped clamping type seedling picking method and parameter optimization
Source: Front Plant Sci. 2023 Jan 12;13:1084563. doi: 10.3389/fpls.2022.1084563 (PMC9878445; doi:10.3389/fpls.2022.1084563)

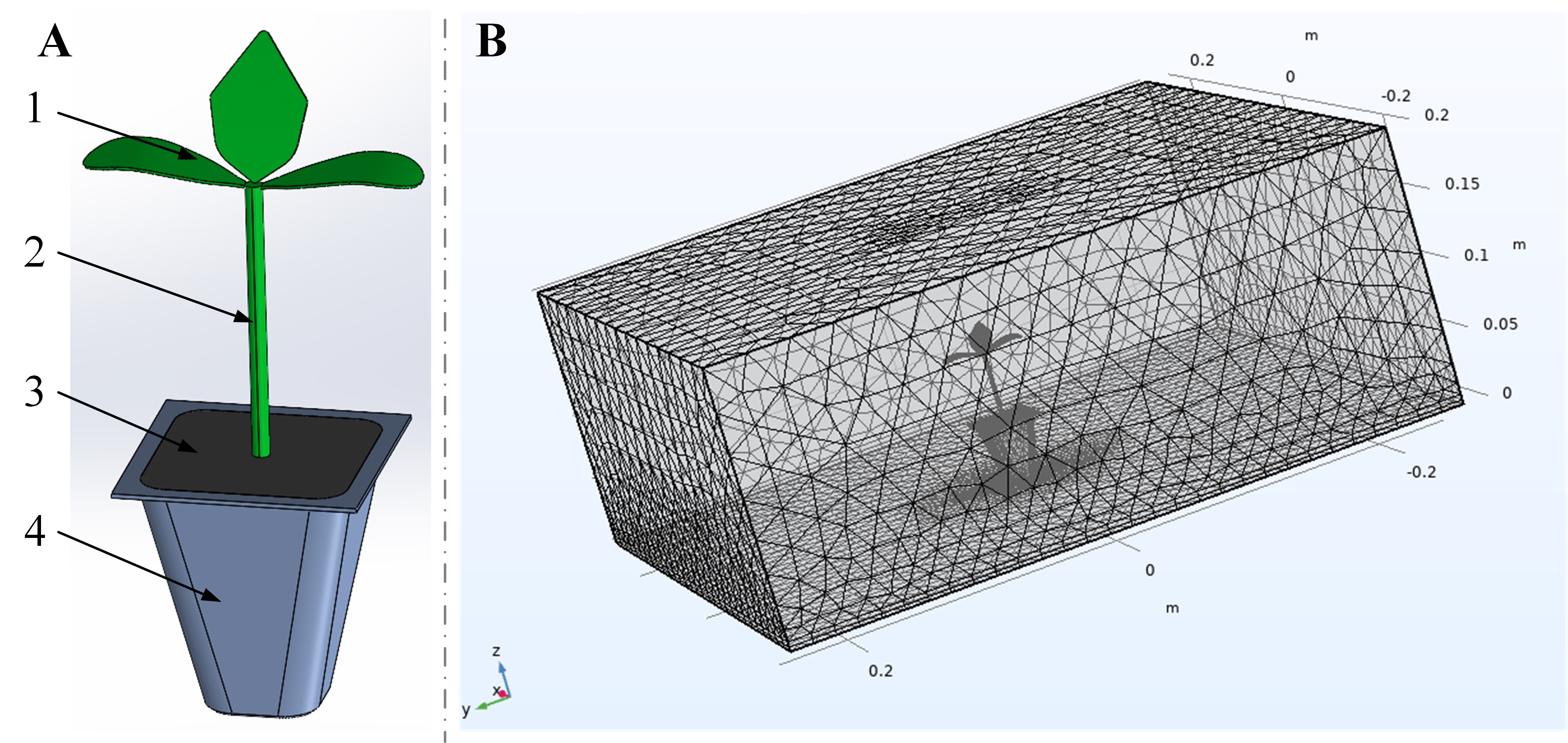

Supplement: Supplementary file 1 [file Image_1.jpeg]

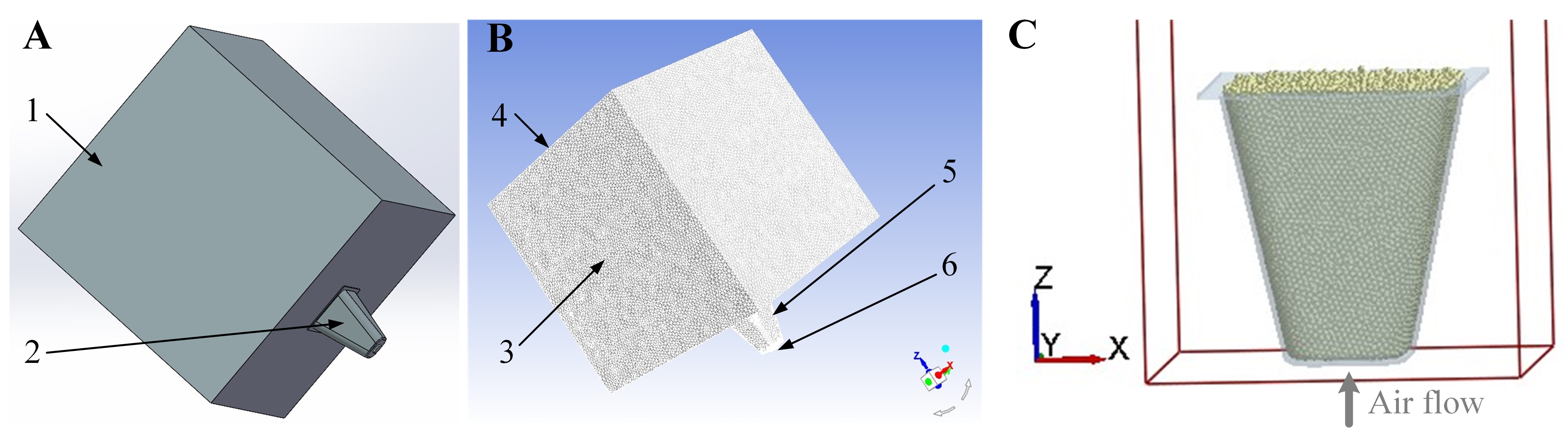

Supplement: Supplementary file 2 [file Image_2.jpeg]

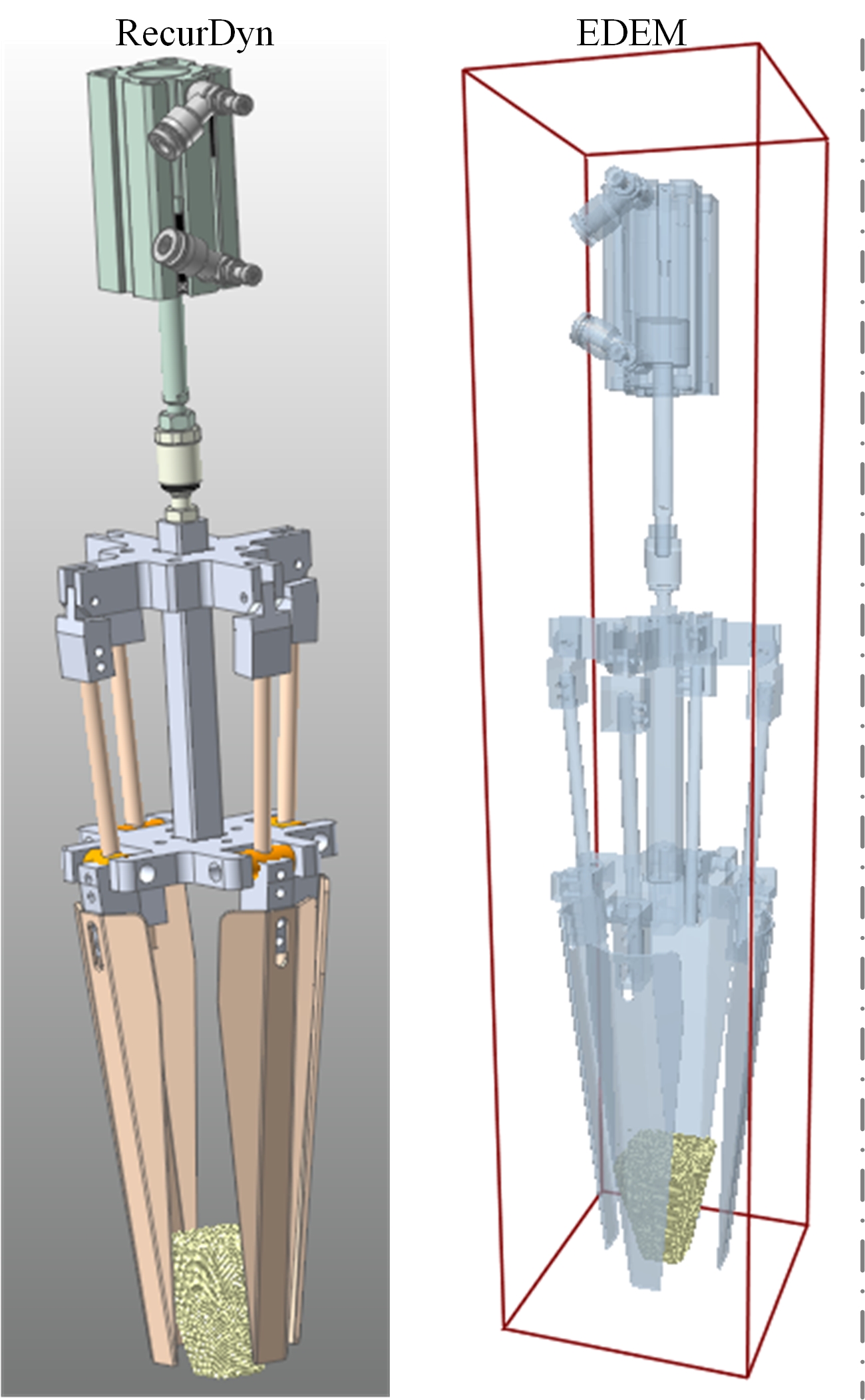

Supplement: Supplementary file 3 [file Image_3.jpeg]

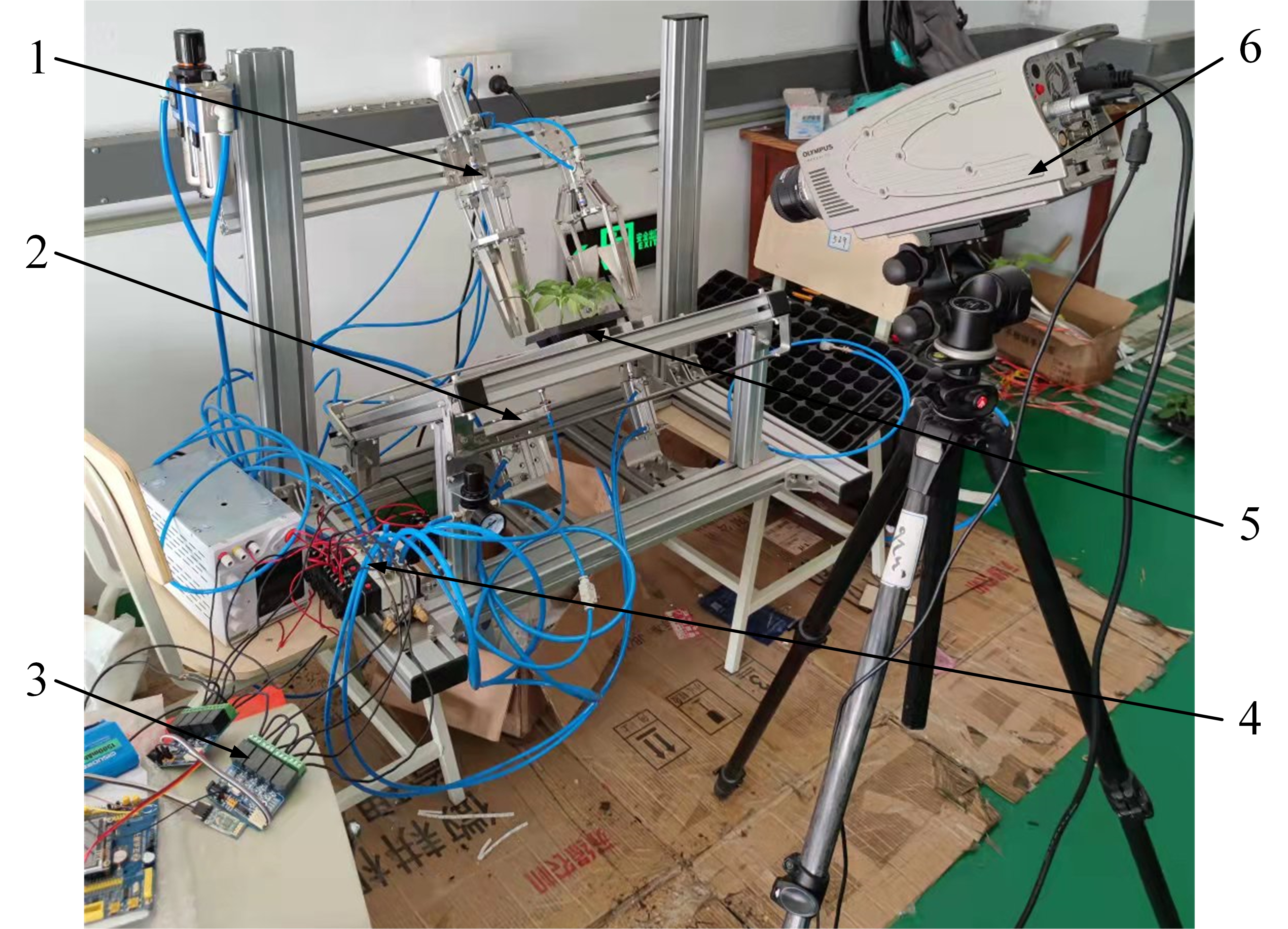

Supplement: Supplementary file 4 [file Image_4.jpeg]
